# Breeding values and index creation for health and behavior traits in Labrador Retriever guide dogs

**DOI:** 10.3389/fvets.2025.1628161

**Published:** 2025-09-10

**Authors:** Joseph A. Thorsrud, Katy M. Evans, C. Kyle Quigley, Krishnamoorthy Srikanth, Antonio Reverter, Laercio R. Porto-Neto, Heather J. Huson

**Affiliations:** ^1^Department of Animal Sciences, Cornell University College of Agriculture and Life Sciences, Ithaca, NY, United States; ^2^The Seeing Eye Inc., Morristown, NJ, United States; ^3^School of Veterinary Medicine and Science, University of Nottingham, Loughborough, United Kingdom; ^4^CSIRO Agriculture & Food, Queensland Bioscience Precinct, Brisbane, QLD, Australia

**Keywords:** Labrador Retrievers, breeding values, guide dog, GBLUP, selection index

## Abstract

**Introduction:**

Genomic breeding values and multi-trait selection indices have significantly advanced genetic improvement in livestock but remain underutilized in guide dog breeding. This study developed a genomically informed selection framework for a population of Labrador Retrievers by integrating health (e.g., dental, ocular, and dermatological conditions) and behavioral (e.g., trainability, distraction level, pace) traits into a “Behavior Score,” “Health Score,” and “Total Score” index by applying Genomic Best Linear Unbiased Prediction (GBLUP) to estimate breeding values.

**Results:**

Phenotypic and genotypic data were collected from 844 dogs over 26 years at The Seeing Eye guide dog school. Predictive performance was evaluated via five-fold cross-validation and correlation-based metrics. Results showed that some dentition related health traits exhibited moderate to high Area Under Receiving Operating Characteristic (AUROC) values (0.79–0.87), indicating potential for immediate use for genetic improvement. In contrast, most other health traits demonstrated weak to moderate predictive accuracy. Behavioral traits exhibited lower predictive accuracy but showed a stronger association with training success. Models were commonly unable to correctly classify individuals for binary or ordinal traits yet performed well in ranking individuals, likely due to lower heritability or strong environmental influences of traits or limitations of the dataset itself. The behavior-focused Total Score (AUROC ~0.72) outperformed health-based indices as a fixed effect in predicting breeding success despite the weaker predictive ability of individual behavioral traits. Incorporating parental scores as fixed effects modestly improved breeding values for success, indicating the importance of integrating additional data sources where available.

**Discussion:**

While these findings underscore the utility of genomic selection for guide dog breeding, they also highlight constraints stemming from small, genetically homogeneous populations and variable phenotyping. Ultimately, we provide the first usable individual and multi-trait genomic approaches to enhance both health and performance outcomes in working dog programs and a foundation to expand upon the reference population and behavioral trait assessment to improve prediction accuracy in the future.

## 1 Introduction

Selective breeding has long been a cornerstone of genetic improvement in domesticated animals, with the dairy cattle industry leading the way in developing sophisticated selection indices that integrate multiple economically and functionally important traits. In species such as dairy cattle, indices such as Net Merit, developed by the United States Department of Agriculture (USDA), have significantly enhanced genetic gain by simultaneously optimizing traits such as milk yield, fertility, longevity, and disease resistance (1, 2). These indices use weighted selection to balance productivity with sustainability. Breeders may apply them to make informed decisions that maximize long-term efficiency and performance with selection weights derived from either economic importance or based on the perceived importance of genetic gain in the desired trait (2, 3). Despite the success of multi-trait selection in livestock breeding, its systematic application in canine breeding programs, particularly those focused on working dogs, remains largely underdeveloped.

In guide dog breeding, genetic selection faces unique challenges (4). Unlike livestock, where economically valuable traits are more directly quantifiable, the success of a guide dog depends on behavioral attributes such as trainability, temperament, and response to environmental stimuli, which must be assessed at multiple developmental stages and are less economically defined (5). Historically, selection in guide dog programs has relied heavily on subjective trainer evaluations and pedigree-based breeding strategies. This has limited genetic progress due to incomplete or inconsistent data collection that cannot account for differences in genetic inheritance not captured by pedigrees (6). An increasing number of guide dog organizations have adopted estimated breeding values (EBVs) to improve selection accuracy with The Seeing Eye being the first to implement them in the early 1980s. The integration of EBVs and genomic selection methodologies offers an opportunity to improve the precision and efficiency of breeding decisions by leveraging genetic information to enhance selection accuracy.

One of the most notable applications of pedigree EBVs in canine breeding has been the long-term effort to reduce the prevalence of hip and elbow dysplasia in working dogs (7–9). Since the 1980s, incorporating EBVs into selection decisions has substantially reduced hip dysplasia rates (7–9). Despite this success, the broader adoption of genomic selection models in working dog breeding has been hindered by several challenges, including small population sizes, varied breeding goals, and limited standardization of trait measurements (10). Additionally, while traditional EBVs have provided genetic improvement, they remain limited by their reliance on pedigree-based relationships rather than genomic data, reducing selection accuracy compared to methods that incorporate direct genomic information.

Genomic Best Linear Unbiased Prediction (GBLUP) has emerged as a powerful tool to generate genomic estimated breeding values (gEBV) across domesticated species by replacing traditional pedigree-based relationship matrices with genomic relationship matrices derived from single nucleotide polymorphism (SNP) markers (11, 12). By incorporating genomic data, GBLUP enhances the accuracy of breeding value estimation, facilitates earlier selection decisions, and accelerates genetic progress. While extensively validated in livestock breeding, where it has revolutionized genetic selection in dairy cattle and poultry, its potential application in guide dog breeding remains largely unexplored with fewer examples of utilization (6, 12, 13). Given its ability to refine selection strategies and improve genetic gain, integrating GBLUP into guide dog breeding programs represents a significant opportunity to enhance health and behavioral outcomes.

A multi-trait selection index tailored to guide dogs would allow breeders to evaluate multiple key traits at the same time, reflecting methods proven effective in livestock breeding. Rather than evaluating individual traits in isolation, a total performance index incorporating health and behavioral metrics would provide a holistic framework for ranking and selecting breeding candidates (4). This approach would promote genetic progress and streamline selection decisions by condensing complex genomic and phenotypic information into a single, interpretable index.

This study aims to develop and apply a performance index for a guide dog breeding population by integrating genomic selection methodologies, specifically GBLUP. By constructing a Total Score that combines health and behavioral measures, we seek to establish a scientifically rigorous, multi-trait selection framework to improve the accuracy of breeding value estimates. We evaluate the predictive power of genomic selection models in identifying key genetic contributors to guide dog success and assess their potential to enhance breeding efficiency. By leveraging genomic technologies and multi-trait selection models, this research aims to provide a data-driven foundation for optimizing genetic selection in guide dog programs, ultimately improving working dogs’ health, performance, and success rates.

## 2 Materials and methods

This study used 26 years of phenotypic and genomic data from The Seeing Eye, Inc. (NJ, United States), a nonprofit that breeds and trains guide dogs. The Seeing Eye breeds and trains German Shepherds, Golden Retrievers, Labrador Retrievers, and Labrador-Golden Retriever crosses. Only Labrador Retrievers were included in this study to ensure a standardized approach to genetic evaluation and selection index development. The dataset included 844 Labrador Retrievers, forming the largest available group with both phenotypic and genotypic records. All data were collected routinely as part of standard operations at The Seeing Eye and did not involve new sample collection or protocols requiring Institutional Animal Care and Use Committee approval.

### 2.1 Phenotypic data collection

Phenotypic data were collected through the puppy raising and training phases from birth until approximately 4.5 years of age. Upon completion, staff classified dogs as either successful guide dogs or failures based on whether they were selected for guide work or breeding (success) or not (failure). An in-house veterinary team recorded health conditions while professional trainers assessed behavioral traits. Dogs missing either phenotypic or genotypic data were excluded to ensure data integrity. The final dataset included 844 Labrador Retrievers with complete data. It also included a subset of 172 dogs whose parents also had full records available which enabled fixed-effect modeling using parental phenotypes. Since behavioral data were scored during the mid-training blindfold test (about 6–8 weeks into guide dog training), all included dogs had cleared early health screening. Thus, the dataset excludes severe early onset conditions and may underestimate health trait effects due to prior removals.

### 2.2 Health trait assessment

Staff diagnosed health phenotypes through standardized physical examinations at four developmental milestones: a puppy physical at approximately 5 weeks of age; a pre-training physical at 14–16 months; and, depending on the dog’s career path, either a pre-breeder physical approximately 3 months after the pre-training physical for those selected as prospective breeders, or a pre-class physical approximately 4 months after the pre-training physical for those not selected as breeders. Each health trait was classified using a predefined diagnostic coding system with a team of five veterinarians independently reviewing all diagnoses. Consensus discussions by the veterinary team allowed for complex cases to maintain diagnostic consistency.

Health conditions were grouped into several categories. Dental conditions included supernumerary teeth, retained deciduous teeth, anodontia, mandibular distocclusion, mandibular prognathism, mandibular mesiocclusion, base narrow mandibular canines, and malocclusion class I. Ocular conditions consisted of distichiasis and persistent pupillary membranes (PPM). Dermatological conditions included histiocytoma, atopic dermatitis, allergic dermatitis, alopecia at the bridge of the nose, acral lick dermatitis, and muzzle folliculitis. Neurological and musculoskeletal conditions included loose interphalangeal collateral ligaments and panosteitis. Other conditions included umbilical hernia and the infectious disease oral papillomatosis. For multi-trait index development, individual counts for dental, ocular, and dermatological conditions were summed to generate Dental Count, Ocular Count, and Dermal Count scores.

### 2.3 Behavior evaluation and scoring

Each dog underwent a comprehensive evaluation based on several behavioral and performance characteristics which were rated using standardized scales. These included Trainability, Rating, Soundness, Distraction, Control Level for Instinct (Neck), Pace, and Pull Strength.

Trainability scores were assigned to all dogs in the study using standardized assessment procedures. A single trainer assigned scores from 1983 until their retirement in 2007, after which a new trainer took over and has remained responsible for scoring to the present day. This continuity helped minimize inter rater variability because only one scorer change occurred over the 26-year period. Both scorers followed established guidelines, and informal harmonization was achieved through shadowing and internal documentation. Still, the possibility of slight drift in scoring interpretation over time cannot be ruled out.

Trainability assessments were conducted at different stages depending on the dog’s progression through the program. Dogs that completed training were scored as they approached their first class. Dogs rejected earlier received their trainability rating at the time of rejection, and breeding dogs were rated upon completion of training. For dogs with multiple assessments, the final available score for each individual was used in the analyses to ensure consistency.

Rating: This scale assessed the dog’s ability to be controlled verbally and physically by handlers of varying skill levels. Ratings ranged from 1 (highly manageable with minimal distraction, suitable for weak handlers) to 5 (high instinct levels, requiring a competent handler).

Soundness: This measure evaluated the dog’s confidence and reaction to environmental stimuli, determining its ability to handle the stress of guide work. Ratings ranged from Sound (fully confident in all environments) to Unsound (lacking the confidence necessary for guide work, with no potential for improvement) across a seven-point scale with the present data containing four levels.

Distraction Level: The extent to which a dog’s instincts interfered with its ability to focus on guide work was recorded. Scores ranged from No Distraction (extremely rare, nearly machine-like focus) to High Distraction (instincts too strong to allow consistent guide work) across a six-point scale with four levels present in the population.

Neck (Control Level for Instinct): This category assessed the physical control necessary to manage the dog when it became distracted. Ratings ranged from Soft (highly sensitive, requiring minimal control) to Hard (useable but only by the strongest handlers) across a five-point scale.

Pace: The dog’s average walking speed was recorded in miles per hour (MPH), classified into five categories by The Seeing Eye for evaluation: Slow: ≤2.0 MPH, Less than Average: 2.0–2.4 MPH, Average: 2.5–3.2 MPH, High Average: 3.3–3.9 MPH, Above average: ≥4.0 MPH.

Pull Strength: The force exerted by the dog while in harness was measured in pounds and categorized as: Light: <2.5 lbs., Less than Average: 2.5–5 lbs., Average: 5–7.5 lbs., Above Average: 7.5–10 lbs., Hard: >10 lbs.

These standardized ratings provided a structured approach to evaluating each dog’s suitability for guide work and its optimal handler match. All ratings were then converted into numeric values based on the trait distributions in Table 1 to allow for consistent model combinations and performance evaluations. Pace, pull, and neck scores were condensed to allow for an ordinal scale due to the selection toward the mean so distinction between high, low, and average was needed with groups of at least 5% of the total population. For distraction and soundness, the traits were converted to binary and ordinal due to the unidirectional selection of those traits.

**Table 1:** Behavior trait standardization.

Trait and trait categories	Count of animals*	Score	Count of animals*
Pace			
Fast	2	2	249
Above average	42		
High average	205		
Average	551	1	551
Less than average	44	0	44
Distractibility			
High	20	1	384
Above average	364		
Average	412	0	460
Less than average	48		
Pull			
Hard	3	2	49
Above average	46		
Average	511	1	744
Defined average	233		
Less than average	51	0	51
Neck			
Hard	9	2	273
Above average	264		
Average	496	1	496
Less than average	69	0	75
Easy	6		
Rating			
5	8	7	8
4+	10	6	10
4	174	5	174
4−	11	4	11
3+	305	3	305
3	282	2	282
3−	32	1	32
2+	22	0	22
Soundness			
Sound	262	2	262
Above average	484	1	484
Average	92	0	98
Less than average	6		

*The count of animals corresponds to the number of animals within that category based on the original and updated scores.

### 2.4 Genotyping and quality control

The Seeing Eye routinely collects whole-blood samples for DNA extraction and storage in its biobank. DNA extraction followed standard Qiagen PureGene protocols with in-house buffers. Quality control and quantification ensured DNA integrity prior to genotyping. Genotyping was conducted using three SNP arrays: the EMBARK (Embark Veterinary Inc. Boston, MA, United States) (Illumina microarray), a 220 K Illumina (Illumina Inc., San Diego, CA, United States) microarray chip, and a 173 K Illumina microarray chip. Datasets were merged, resulting in an initial dataset of 239,478 SNPs across 2,176 dogs, including dogs across three breeds and without complete phenotypic records, with an overall genotyping rate of 91.4%. SNP quality control was performed using PLINK v1.9, applying genotype call rate thresholds of less than 90%, sample call rates below 90%, Hardy–Weinberg equilibrium at *p* < 1 × 10^−5^, and minor allele frequencies less than 0.01. After quality filtering, 166,463 SNPs remained for imputation (14).

Phasing and imputation were conducted using Beagle v5.2 (15), leveraging a reference panel mapped to CanFam 3.1, which included 660 dogs across 157 modern breeds and village dogs, including 23 Labrador Retrievers (16–19). The final dataset, pruned for linkage disequilibrium, contained 1,219,623 SNPs. A reduced subset of 220 K SNPs corresponding to the Illumina chip was retained for the 844 dogs with full phenotypic data in the final GBLUP analysis to allow for easy comparison to existing SNP datasets.

### 2.5 GBLUP model evaluation

An additive Genomic Relationship Matrix (GRM) was constructed using SNP data to quantify the genetic relatedness among individuals, and all analyses were conducted with a mixed-model association approach using a Restricted Maximum Likelihood (REML) algorithm to estimate variance components. The GBLUP model used to obtain the gEBV included the fixed effects of birth year and the first three principal components as linear covariates (20). Model performance was evaluated through five-fold cross-validation, partitioning data into five subsets, training on four, and validating the remaining subset. Through this, each individual was a part of the testing dataset for a fold and had a predicted and actual phenotype that could be compared. In addition, pseudo-heritability was calculated for each trait utilizing Golden Helix SNP and Variation Suite SNP Analysis v8.9.1 (RRID: SCR_001285) (Golden Helix, Inc., Bozeman, MT, United States).^1^

Multiple metrics were employed to assess prediction accuracy. These included the Matthews Correlation Coefficient (MCC) and Area Under the Receiver Operating Characteristic Curve (AUROC) for binary traits, Kendall’s Tau and Spearman’s Rho for ordinal traits, and Pearson’s Correlation (R) and the normalized mean squared error (NMSE) for continuous traits.

MCC measures the quality of binary classifications and accounts for true positives (TP), false positives (FP), true negatives (TN), and false negatives (FN) in a single statistic. Values of MCC range from −1 (complete misclassification) to +1 (perfect classification), with 0 indicating a result no different than random guessing.

AUROC quantifies how well the model differentiates between classes by plotting the true positive rate (sensitivity) against the false positive rate (1 − specificity). AUROC scores generally range from 0.5 (no different than random) to 1.0 (perfect discrimination). Scores below 0.5 indicate worse-than-chance performance.

Kendall’s Tau and Spearman’s Rho are rank-based correlation coefficients that measure ordinal and monotonic relationships ranging from −1 to +1, with 0 indicating no relationship.

Pearson’s Correlation (R) quantifies the linear relationship between predicted and observed values, ranging from −1 to +1. A value of 0 indicates no linear correlation, while ±1 denotes a perfect relationship.

Normalized Mean Squared Error (NMSE) is a metric that evaluates how well a model’s predictions match the actual data. This approach is similar to Mean Squared Error (MSE) but adjusted for the scale of the data. While MSE measures the average squared difference between predicted and actual values, NMSE divides this error by the variance of the actual data, making it easier to compare across datasets with different scales. An NMSE value closer to 0 indicates more accurate predictions, while higher values suggest greater prediction error.

### 2.6 Multi-trait index creation

Composite indices were developed to systematically capture guide dogs’ overall health and behavioral suitability. These indices were the Health Count, Dental Count, Ocular Count, Dermal Count, Behavior Score, Health Score, and Total Score, each designed to integrate relevant traits into a single standardized metric.

The health counts represent the total number of diagnosed health conditions for each dog. Conditions were categorized as dental (for instance, supernumerary and retained deciduous teeth, enamel defects, and various malocclusions), ocular (including distichiasis, retinal folds and dysplasia, corneal cholesterolosis, iris cyst, and persistent pupillary membrane), dermatological (such as histiocytoma, atopic and allergic dermatitis, alopecia on the bridge of the nose, acral lick dermatitis, and muzzle folliculitis), neurological (laryngeal paralysis), congenital defects (umbilical hernia), musculoskeletal (panosteitis and loose interphalangeal collateral ligaments), and viral/infectious (oral papillomatosis). Each diagnosed condition contributed one point to the individual’s Health Count. The Dental Count captures only dental anomalies. All recorded dental conditions added to the total Dental Count for that individual. The Ocular Count reflects the number of ocular abnormalities. Each condition detected in an individual added one point to the Ocular Count. The Dermal Count is the total of all dermatological conditions present in an individual.

The Behavior Score was derived by normalizing each behavioral trait according to its relationship with guide dog success. A transformation was used for traits negatively correlated with success, including neck, distraction, and rating, so higher raw values produced lower normalized scores using Equation 1. Traits positively correlated with success (soundness and trainability) were normalized with retained directionality using Equation 2. Traits with an optimal intermediate value (pace and pull) were normalized to reflect how far each raw score deviated from the ideal point using Equation 3. Each trait was assigned a weight based on its correlation with success, and the overall Behavior Score was calculated as the weighted sum of these transformed values. This resulted in scores between 0 and 1 for each trait, with 1 being closer to optimal and 0 negatively correlated to success. The values were then multiplied by the Pearson correlation for that trait with success and summed.


(1) Negative Behavior Normalized=∣Xmax−Xindividual∣÷Xmax

(2) Positive Behavior Normalized=Xindividual÷Xmax

(3) Neutral Behavior Normalized=1−∣Xindividual−1∣

The Health Score was constructed by normalizing each health-related measure to ensure comparability. This was done by multiplying the binary status with the correlation to success for that trait. Summing these normalized trait values provided a composite Health Score that captured each dog’s overall severity of health conditions. All trait correlations to success are listed in Table 2.

**Table 2:** Trait correlations to success.

Trait	Correlation to success
Base narrow mandibular canines	−0.026
Malocclusion class I	−0.035
Distichiasis	−0.032
PPM	−0.025
Loose interphalangeal collateral ligaments	−0.003
Umbilical hernia	−0.009
Histiocytoma	−0.036
Atopic dermatitis	−0.1
Allergic dermatitis	−0.009
Alopecia bridge of nose	−0.027
Acral lick dermatitis	−0.029
Muzzle folliculitis	−0.009
Panosteitis	−0.02
Oral papillomatosis	−0.02
Supernumerary teeth	−0.024
Retained deciduous teeth	−0.013
Anodontia	−0.014
Mandibular distocclusion	−0.105
Mandibular prognathism	−0.031
Mandibular mesiocclusion	−0.047
Distraction	−0.107
Neck	−0.108
Soundness	0.127
Rating	−0.144
Pace	0.02
Pull	0.017
Trainability	0.531
Health score	−0.02
Behavior score	0.324

Finally, the Total Score was determined by combining the Health Score and Behavior Score into a single weighted index using Equation 4: w_health_ and w_behavior_ are weighting factors derived from correlation analyses linking these traits to guide dog success. This combined score ensures that health and behavioral traits are appropriately emphasized in selection decisions.


(4) $$\begin{array}{l} \text{Total Score}=w_{\text{health}}\times (\text{health score})+w_{\text{behavior}}\\ \times (\text{behavior score}) \end{array}$$

A subset of animals also had complete phenotypic records on both parents in the dataset. These 172 animals were used as a subset to determine the utility of parental phenotypes as fixed effects on the breeding value performance of the offspring. The indices used were Parent Health Score, Parent Behavior Score, and Parent Total Score as a fixed effect for both Total Score and Success breeding values.

## 3 Results

The performance of estimated breeding values (EBVs) and selection indices was evaluated across multiple health and behavioral traits using Genomic Best Linear Unbiased Prediction (GBLUP). Predictive accuracy was assessed through Matthews correlation coefficient (MCC), area under the receiver operating characteristic curve (AUROC), Kendall’s Tau, and Spearman’s Rho, with five-fold cross-validation employed for model validation.

### 3.1 Individual traits

**Figure 1:**
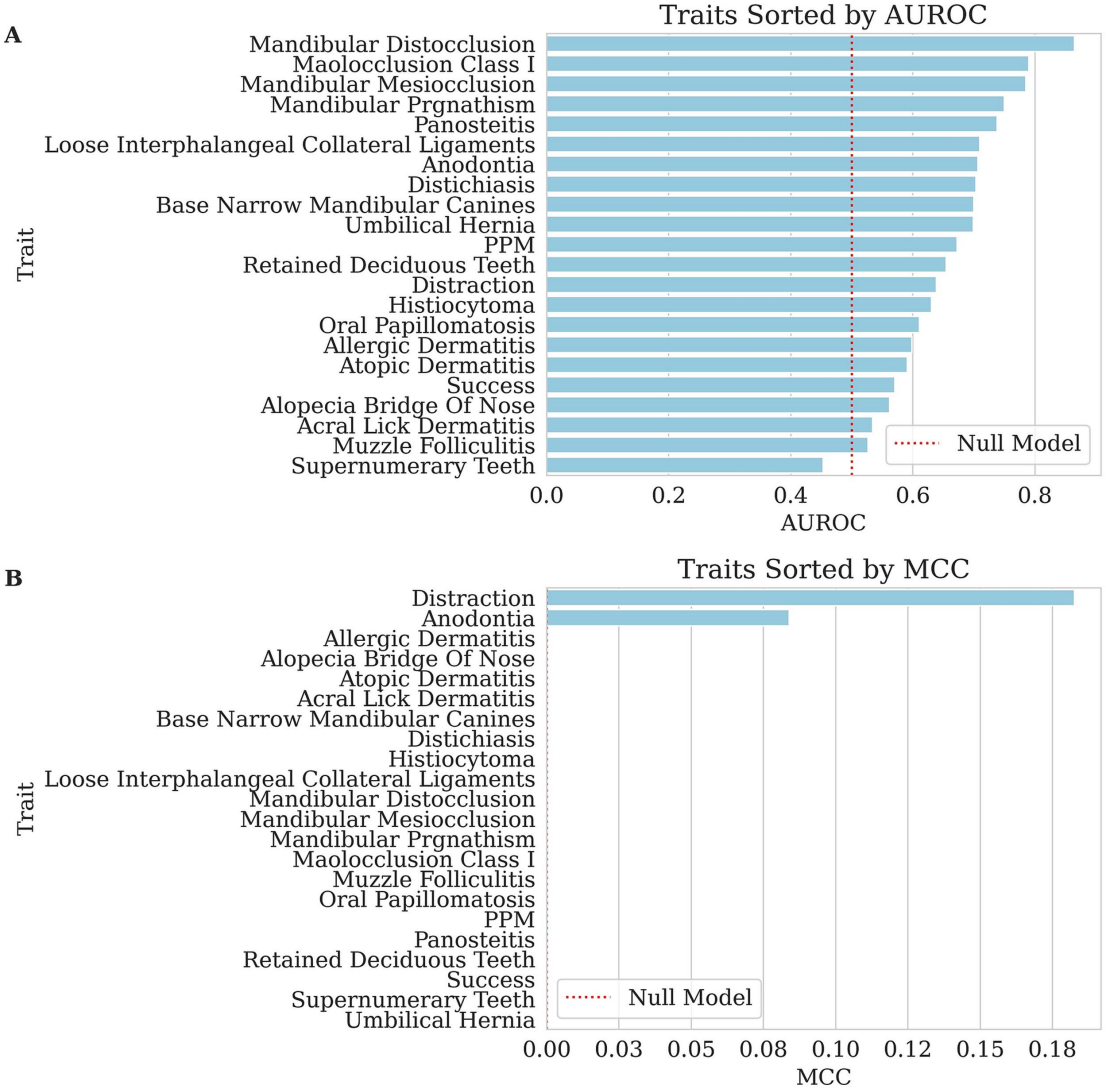
Bar charts of performance for each binary trait’s genomic breeding value prediction, with traits sorted from highest to lowest along the x-axis. The dashed red lines indicate null performance. **(A)** The area under the receiving operating curve (AUROC), where larger values reflect better ranking of affected versus unaffected dogs in GBLUP risk-ranking. **(B)** Matthew’s Correlation Coefficient (MCC), where larger values indicate better discrimination of affected versus unaffected dogs in GBLUP risk-classification. In both panels, traits with higher scores demonstrate greater predictability, whereas lower scores indicate weaker performance for this dataset.

**Figure 2:**
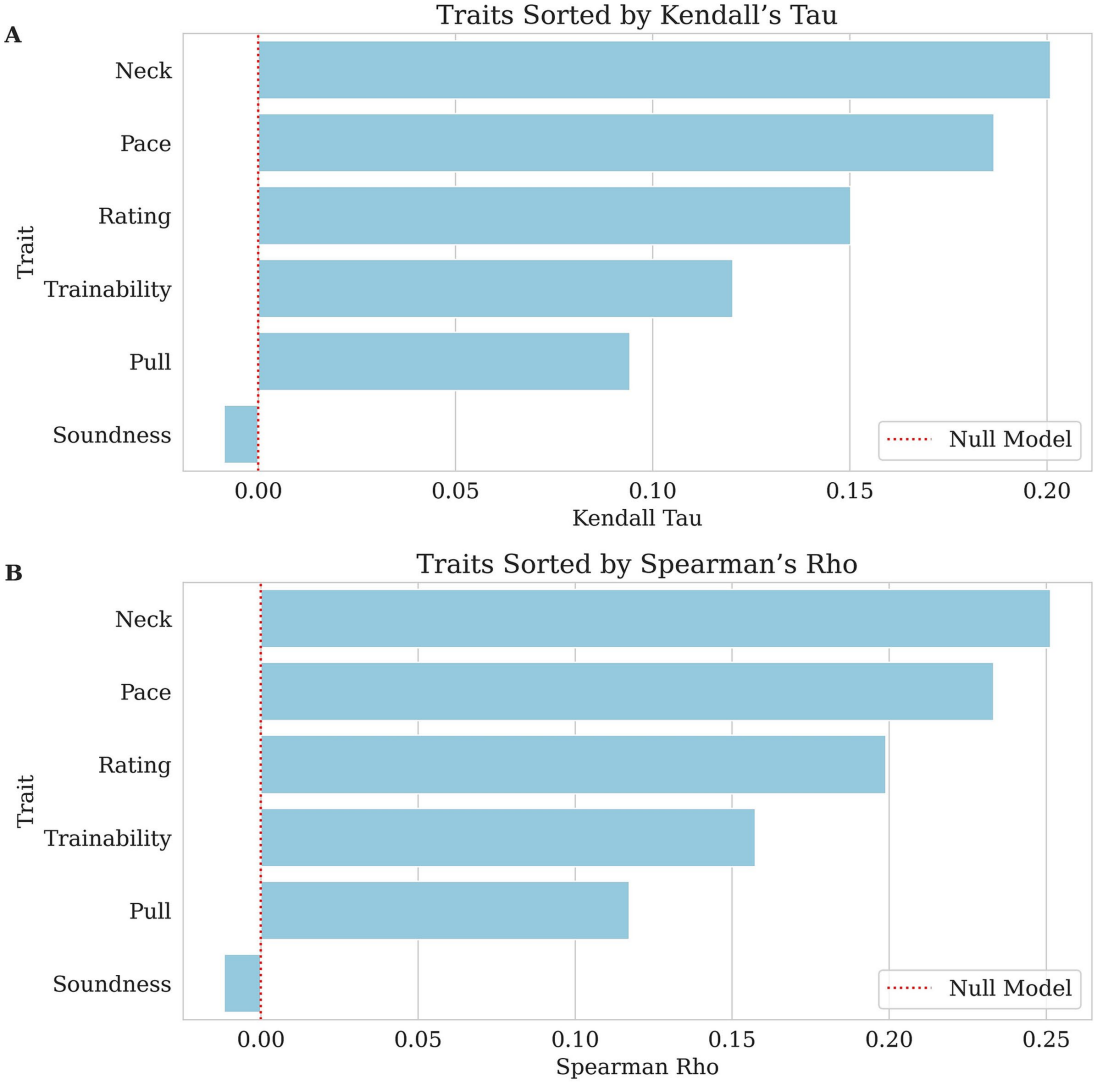
Bar charts of each ordinal trait’s genomic breeding value prediction, with traits sorted from highest to lowest correlation along the x-axis. The dashed red lines indicate null performance. **(A)** Kendall’s Tau values, where larger values indicate stronger agreement between predicted and actual ranking. **(B)** Spearman’s Rho values reflect the correlation between predicted and actual ranking in GBLUP values. In both panels, lower correlation scores indicate weaker predictability for this dataset.

The heritability of the individual trait and the model’s predictive performance were evaluated. The predictive performance was assessed using MCC, AUROC, Kendall’s Tau, and Spearman’s Rho as appropriate based on the data type. The results are summarized in Table 3 and Figures 1, 2.

**Table 3:** Scoring metrics for individual traits.

Trait	Pseudo-heritability	MCC	AUROC	Kendall Tau	Spearman Rho
Base narrow mandibular canines	0.146	0.000	0.700		
Malocclusion class I	0.148	0.000	0.790		
Distichiasis	0.092	0.000	0.704		
PPM	0.142	0.000	0.672		
Loose interphalangeal collateral ligaments	0.109	0.000	0.710		
Umbilical hernia	0.037	0.000	0.699		
Histiocytoma	0.039	0.000	0.631		
Atopic dermatitis	0.044	0.000	0.591		
Allergic dermatitis	0.019	0.000	0.598		
Alopecia bridge of nose	0.022	0.000	0.562		
Acral lick dermatitis	0.046	0.000	0.534		
Muzzle folliculitis	0.023	0.000	0.527		
Panosteitis	0.163	0.000	0.738		
Oral papillomatosis	0.089	0.000	0.611		
Supernumerary teeth	<0.000	0.000	0.453		
Retained deciduous teeth	0.131	0.000	0.655		
Anodontia	0.247	0.084	0.706		
Mandibular distocclusion	0.269	0.000	0.865		
Mandibular prognathism	0.080	0.000	0.750		
Mandibular mesiocclusion	0.139	0.000	0.785		
Success	0.041	0.000	0.570		
Distraction	0.177	0.183	0.639		
Neck	0.209			0.201	0.251
Soundness	<0.000			−0.009	−0.012
Rating	0.179			0.150	0.199
Pace	0.153			0.187	0.233
Pull	0.066			0.094	0.117
Trainability	0.147			0.120	0.158

For most traits, the MCC values were 0.000, indicating poor classification performance. Across the traits, pseudo-heritability estimates were generally low to moderate (median ≈ 0.14), suggesting that while genetic factors contribute, substantial environmental or measurement noise remains. Related, the AUROC values varied across traits, with the highest AUROC observed for mandibular distocclusion (AUROC = 0.865, pseudo-heritability = 0.269), followed by malocclusion class I (AUROC = 0.790, pseudo-heritability = 0.148) and mandibular mesiocclusion (AUROC = 0.785, pseudo-heritability = 0.139). Although MCC remained at 0.000 for these traits, the elevated AUROC scores indicate that the model still provided meaningful risk ranking, even if strict classification thresholds were not optimized. This apparent contradiction arises because MCC and AUROC measure different aspects of model performance. MCC evaluates the quality of binary classifications made at a particular decision threshold, balancing true and false positives and negatives. It is especially sensitive to class imbalance, commonly seen in our traits, and requires the model to make discrete decisions. In contrast, AUROC assesses the model’s ability to discriminate between classes across all possible thresholds, capturing how well the model ranks positive cases above negative ones. Thus, a high AUROC paired with a low MCC indicates that while the model can correctly order cases by risk, it struggles when forced to commit to a binary decision using a single cutoff point.

Among other dentition-related conditions, retained deciduous teeth (AUROC = 0.655, pseudo-heritability = 0.131) and supernumerary teeth (AUROC = 0.453, pseudo-heritability <0.000) had differing levels of classification accuracy and base narrow mandibular canines (AUROC = 0.700, pseudo-heritability = 0.146) also showed moderate AUROC values. Notably, anodontia was the only health trait with an MCC greater than zero (MCC = 0.084, AUROC = 0.706, pseudo-heritability = 0.247), suggesting marginal but the first non-trivial predictive power in proper classification.

For dermatological conditions, histiocytoma (AUROC = 0.631, pseudo-heritability = 0.039), atopic dermatitis (AUROC = 0.591, pseudo-heritability = 0.044), allergic dermatitis (AUROC = 0.598, pseudo-heritability = 0.019), and alopecia of the bridge of the nose (AUROC = 0.562, pseudo-heritability = 0.022) had moderate AUROC values, indicating weak but non-random ranking performance. However, conditions such as muzzle folliculitis (AUROC = 0.527, pseudo-heritability = 0.023) and acral lick dermatitis (AUROC = 0.534, pseudo-heritability = 0.046) exhibited lower discrimination abilities. Distichiasis (AUROC = 0.704, pseudo-heritability = 0.092) and oral papillomatosis (AUROC = 0.611, pseudo-heritability = 0.089) also demonstrated moderate classification performance.

The orthopedic and musculoskeletal conditions also showed variable AUROC performance. Panosteitis (AUROC = 0.738, pseudo-heritability = 0.163) had one of the higher scores in this category, suggesting some predictive value. Loose interphalangeal collateral ligaments (AUROC = 0.710, pseudo-heritability = 0.109), PPM (AUROC = 0.672, pseudo-heritability = 0.142) and umbilical hernia (AUROC = 0.699, pseudo-heritability = 0.037) had slightly lower but notable predictive performance.

The success and distraction traits had mixed results. Success (AUROC = 0.570, pseudo-heritability = 0.041) had a weak classification performance, while distraction (MCC = 0.183, AUROC = 0.639, pseudo-heritability = 0.177) performed slightly better than most traits, and its higher heritability underscores a clearer genetic influence. Other behavioral traits and performance metrics were evaluated using Kendall’s Tau and Spearman’s Rho. The highest correlations were observed for neck (Kendall’s Tau = 0.201, Spearman’s Rho = 0.251, pseudo-heritability = 0.209) and pace (Kendall’s Tau = 0.187, Spearman’s Rho = 0.233, pseudo-heritability = 0.153), suggesting a weak to moderate association between predicted and actual values. Rating (Kendall’s Tau = 0.150, Spearman’s Rho = 0.199, pseudo-heritability = 0.179) and trainability (Kendall’s Tau = 0.120, Spearman’s Rho = 0.158, pseudo-heritability = 0.47) also demonstrated weak but notable correlations. Pull (Kendall’s Tau = 0.094, Spearman’s Rho = 0.117, pseudo-heritability = 0.066) was weaker than the previously mentioned behavioral traits but still showed a minor positive association. However, soundness (Kendall’s Tau = −0.009, Spearman’s Rho = −0.012, pseudo-heritability < 0.000) showed effectively no relationship, indicating poor predictive capability.

### 3.2 Selection indices and fixed effects

The analysis evaluated multi-trait index scores which included different fixed effects. The performance comparisons utilized AUROC, MCC, Kendall’s Tau, Spearman’s Rho, Pearson’s R, and NMSE to quantify the effectiveness of the GBLUP model in index performance.

**Figure 3:**
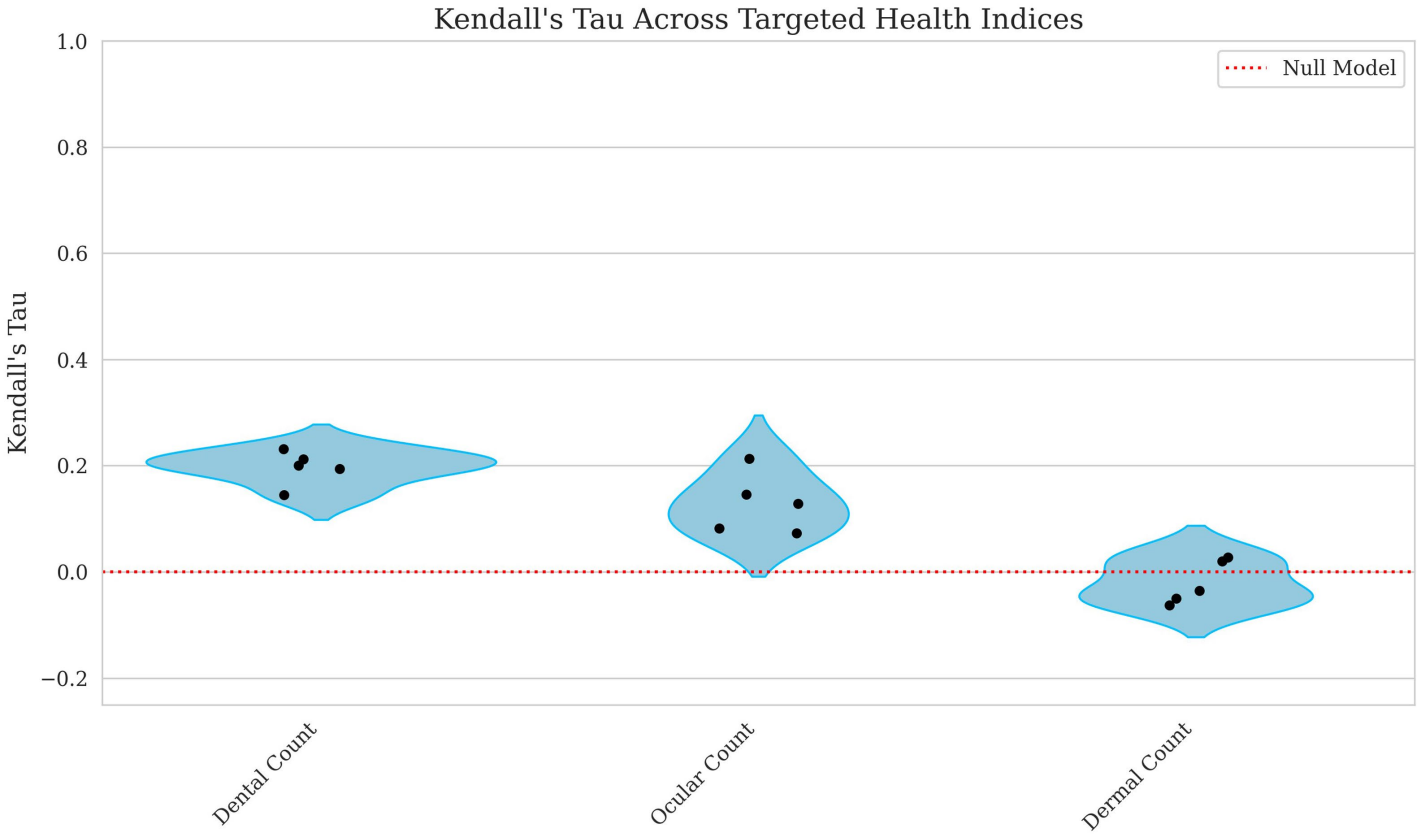
Violin plots of Kendall’s Tau for dental, ocular, and dermal count breeding values. Each point represents a single fold’s score with the red line indicating a null model’s performance. The x axis corresponds to the model performance for dental, ocular and dermal count breeding values with the y-axis corresponding to the Kendall’s Tau score. The lower values correspond to lower predictive ability of the model.

**Figure 4:**
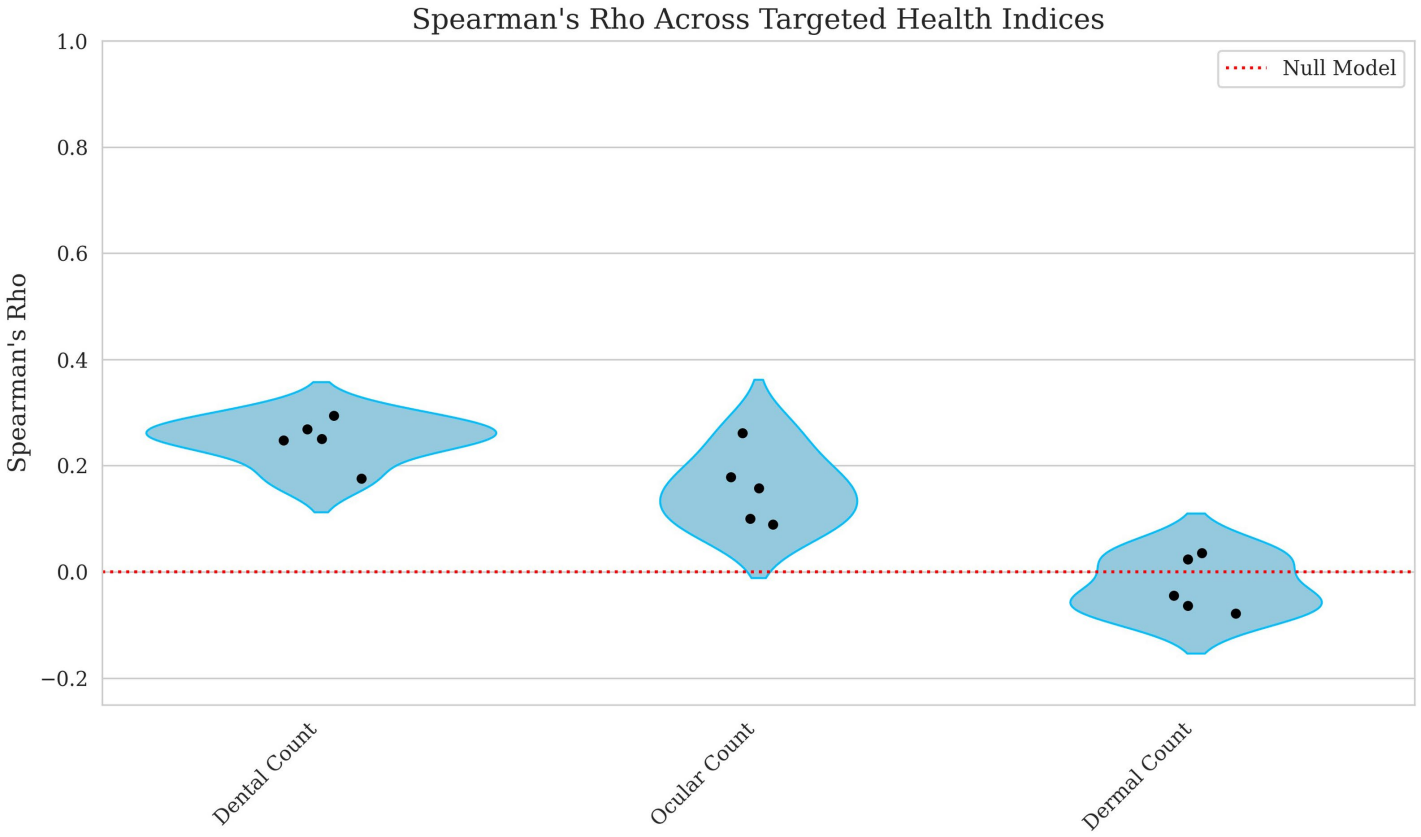
Violin plots of Spearman Rho for dental, ocular, and dermal count breeding values. Each point represents a single fold’s score with the red line indicating a null model’s performance. The x-axis corresponds to the model performance for Dental, Ocular, and Dermal Count breeding values, and the y-axis corresponds to the Spearman’s Rho score. Lower values indicate weaker predictive ability of the model.

The model’s ability to predict count-based indices showed weak to negligible correlations. The dental count had the highest correlation, with Kendall’s Tau = 0.197 and Spearman’s Rho = 0.247, suggesting a weak but notable relationship displayed in Figures 3, 4. Ocular count performed slightly worse, with Kendall’s Tau = 0.129 and Spearman’s Rho = 0.158, indicating a weaker association. Dermal count, exhibited no meaningful correlation with Kendall’s Tau = −0.020 and Spearman’s Rho = −0.025, implying this trait’s lack of predictive accuracy.

**Figure 5:**
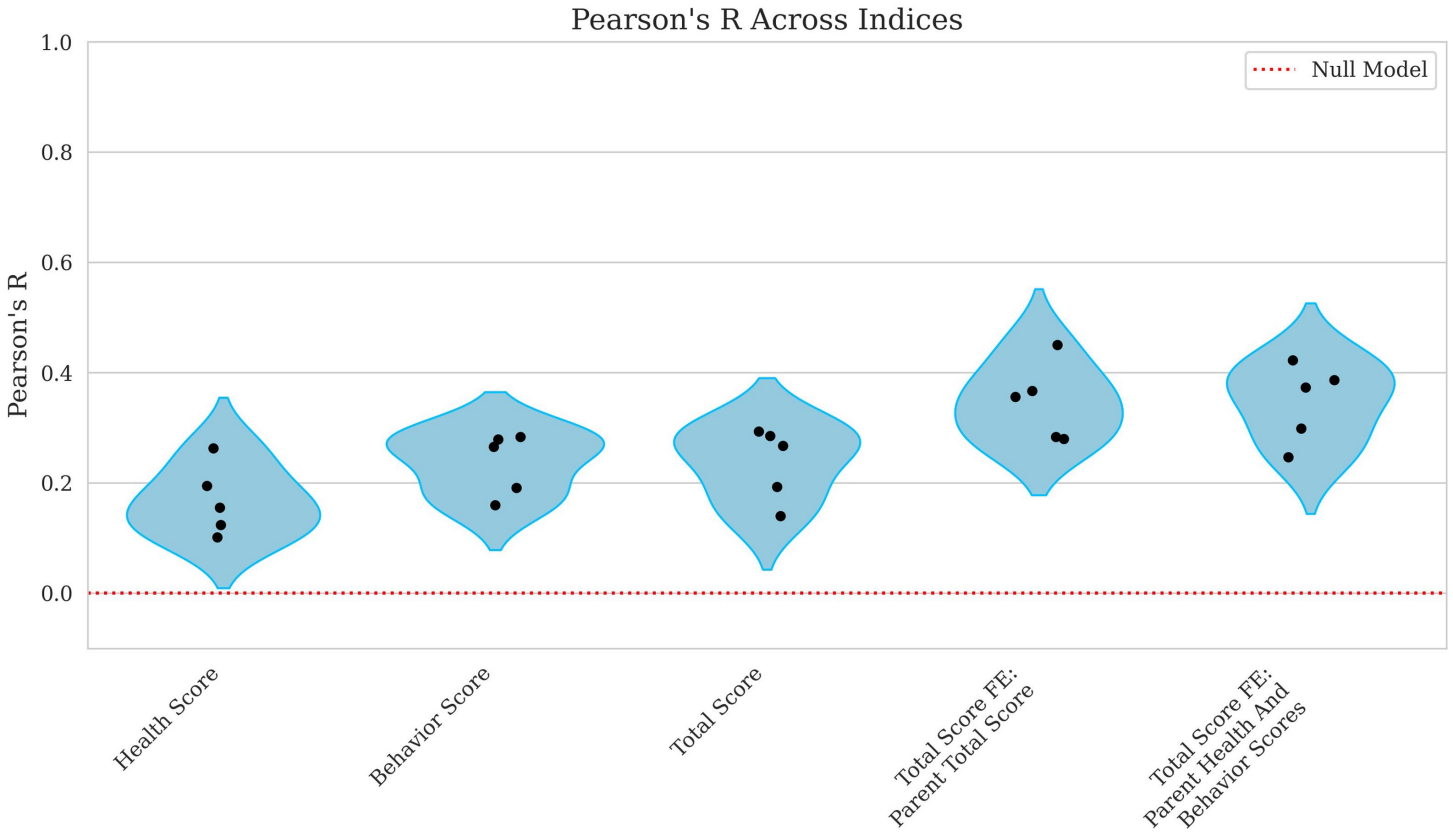
Violin plots of Pearson R for health, behavior, total score, and total score with parental fixed effects breeding values. Each plotted point represents the Pearson correlation for an individual fold within the cross-validation. The x-axis displays the different breeding value models evaluated including Health Score, Behavior Score, and Total Score along with Total Score including Parent Total Score and Total Score including Parent Health and Behavior Scores as fixed effects. The y-axis reflects the corresponding Pearson’s R values, which quantify the linear relationship between predicted and observed scores. A red reference line indicates the expected performance under a null model. Lower correlation values denote weaker predictive performance.

**Figure 6:**
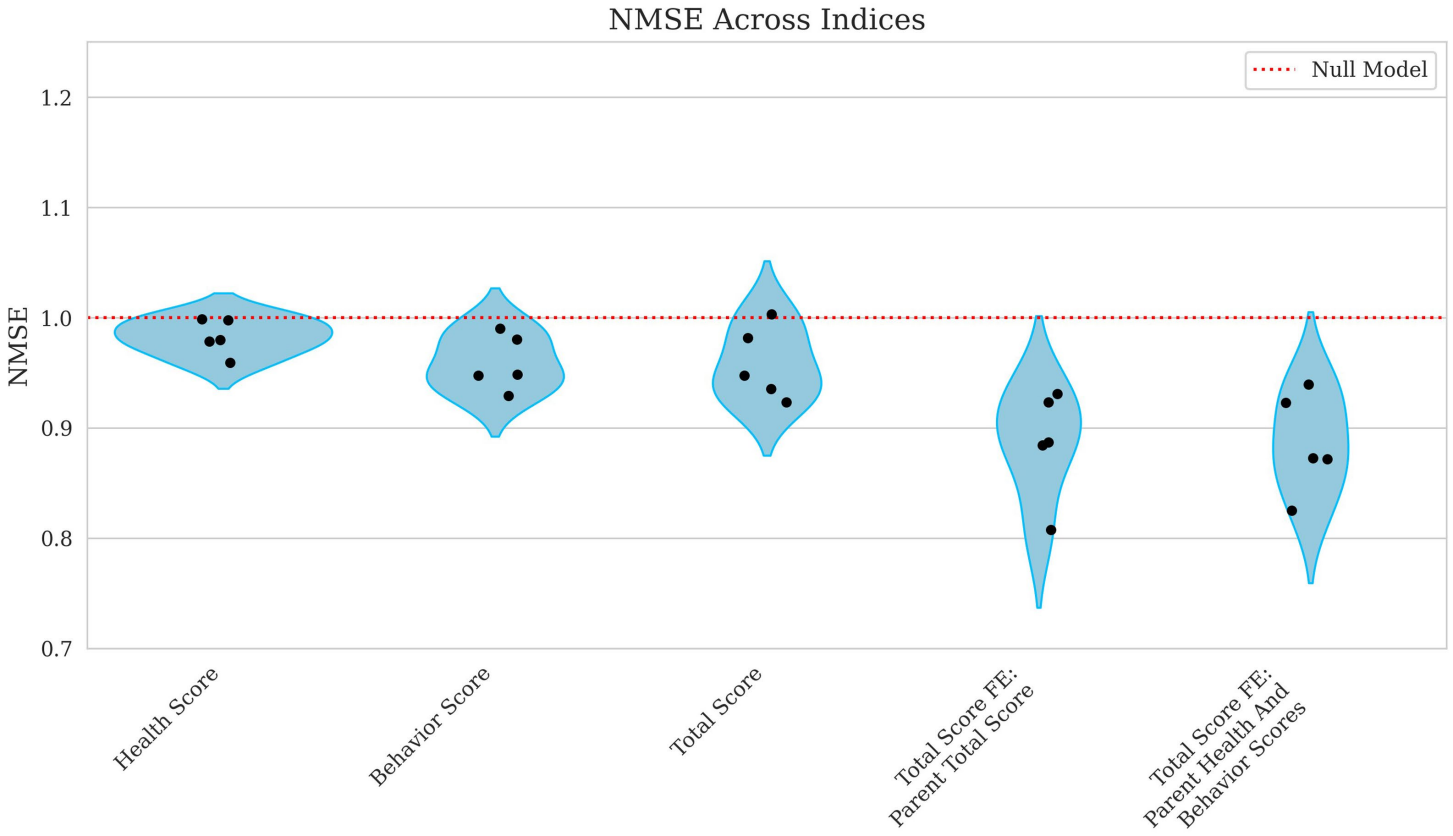
Violin plots of NMSE for health, behavior, total score, and total score with parental fixed effects breeding values. Each plotted point represents the NMSE for an individual fold within the cross-validation. The x-axis differentiates the breeding values for Health Score, Behavior Score, and Total Score, along with Total Score models incorporating Parent Total Score and Total Score incorporating Parent Health and Behavior Scores as fixed effects. The y-axis shows the corresponding NMSE values, which measure the average squared prediction error normalized by the variance of the observed values. A red reference line indicates the expected performance under a null model. Higher NMSE values indicate greater prediction error and thus poorer model performance.

Correlations improved slightly from weak to moderate when evaluating the model’s ability to predict broader performance-based scores. Health score had a low correlation, with Pearson’s R = 0.168 and a high error rate (NMSE = 0.983), as shown in Figures 5, 6, indicating poor predictive capability. In contrast, behavior scores and total scores performed slightly better, with Pearson’s R ~ 0.23 and NMSE ~ 0.96, suggesting a slightly stronger but still weak association.

Incorporating parental phenotypes improved the model’s ability to predict the total score of an individual. Figures 5, 6 show how total score using the parental total score as a fixed effect achieved a Pearson’s R = 0.347 and NMSE = 0.886 while using both parent health score and parent behavior score yielded nearly identical results (Pearson’s R ~ 0.35, NMSE ~ 0.89). These findings suggest that while genetic or inherited factors play a role, their contribution remains modest.

**Figure 7:**
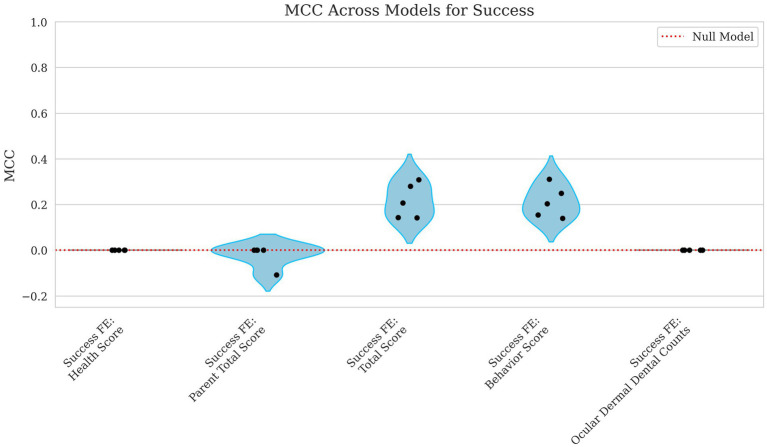
Violin plots of MCC for success with parental total score, total score, behavior score, and ocular, dermal, and dental counts as fixed effects. Each dot represents the MCC from a single fold in the cross-validation process. The x-axis identifies the fixed effects included in the model including Health Score, Parent Total Score, Total Score, Behavior Score, and the Ocular, Dermal, and Dental Counts. The y-axis presents the MCC values, which reflect the model’s ability to correctly classify success outcomes. A red dashed line marks the performance expected under a random model. Lower MCC scores suggest reduced accuracy in binary classification.

**Figure 8:**
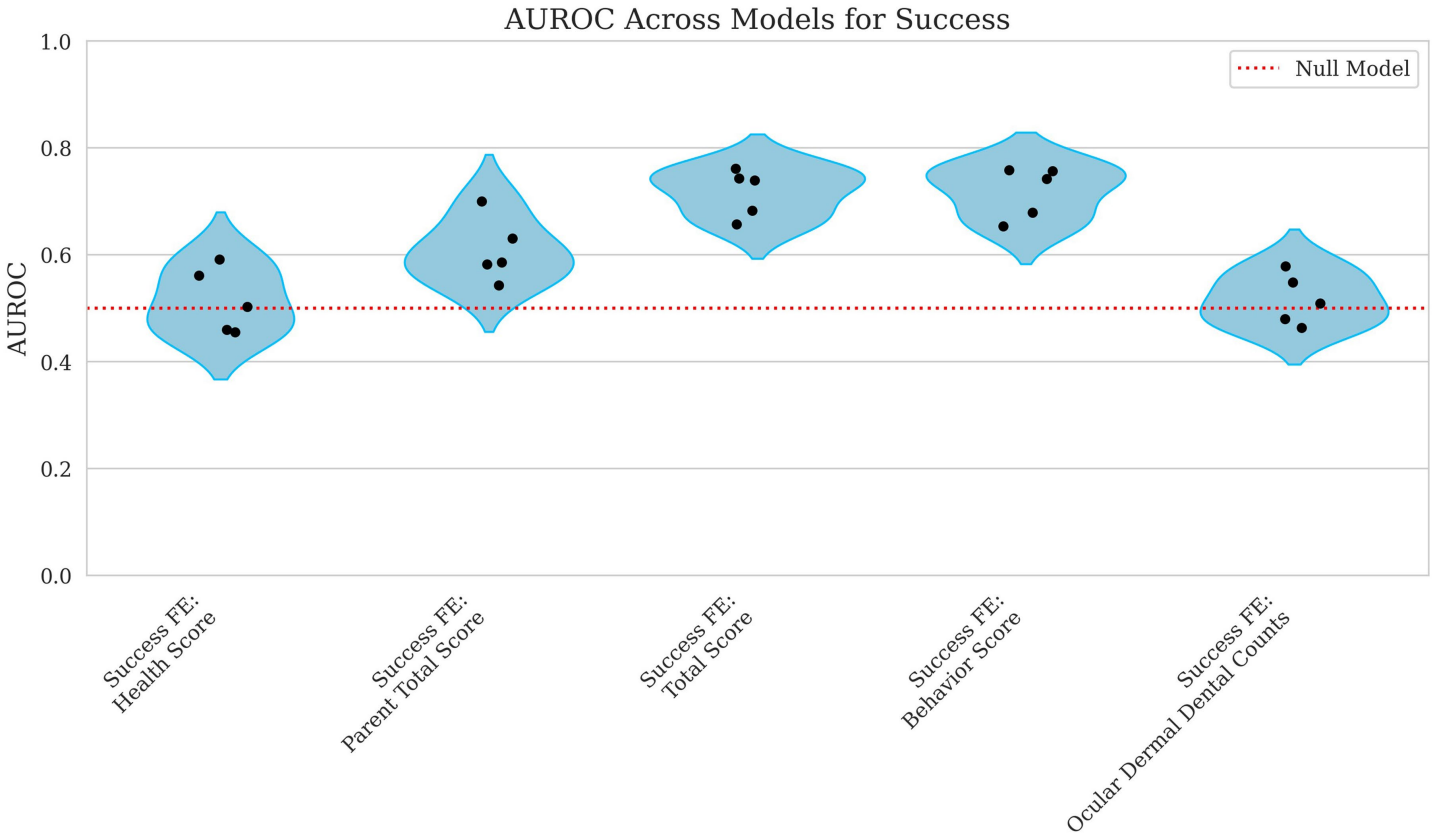
Violin plots of AUROC for success with parental total score, total score, behavior score, and ocular, dermal, and dental counts as fixed effects. Each point represents the AUROC value from a single cross-validation fold. The x-axis corresponds to the fixed effects included in the model including Health Score, Parent Total Score, Total Score, Behavior Score, and the Ocular, Dermal, and Dental Counts. The y-axis displays the AUROC scores, which quantify the model’s ability to distinguish between successful and unsuccessful outcomes across all classification thresholds. A red reference line indicates the expected performance of a random classifier. Lower AUROC values reflect weaker predictive discrimination.

The model’s predictive performance for success varied widely depending on the features used. Using health score as a fixed effect alone resulted in an MCC = 0.000 and AUROC = 0.514, indicating performance no better than chance as shown in Figures 7, 8. However, when behavior score was included, performance improved notably (MCC = 0.212, AUROC = 0.718), suggesting a stronger link between behavioral traits and success in this dataset. Similarly, total score as a fixed effect resulted in MCC = 0.216 and AUROC = 0.716, demonstrating a comparable predictive power level.

When using ocular, dermal, and dental counts as fixed effects, the model again performed at chance level (MCC = 0.000, AUROC = 0.516) displayed in Figures 7, 8, indicating that these factors were not useful in predicting success. Finally, incorporating the parent total score shows a slight decline in performance for MCC and a slight increase in AUROC (MCC = −0.022, AUROC = 0.608), suggesting that parental influence, while somewhat predictive of overall scores, was less useful in predicting success outcomes.

## 4 Discussion

### 4.1 Advantages of genomic estimated breeding values

Historically, breeding values have been predicted from pedigree-derived datasets. However, replacing these with genomic relationship estimates may sharpen that estimate to allow for a more granular view of the genomic architecture. This challenge is exemplified by work using a wheat dataset supplemented with simulations of varying numbers of quantitative trait loci (QTL). The work demonstrated that the performance of different methods including GBLUP depended heavily on the trait’s genetic architecture with model performance altered by the difference between many minor QTL compared to only a few QTL with larger effects. Further illustrating the point, multi-generation simulations demonstrated that genomic estimated breeding values (gEBV) substantially exceeded traditional BLUP for low-heritability traits, provided sufficient training data were collected, particularly in situations such as sex-limited traits or those costly to measure (21). In a long-term field study of Soay sheep, leveraging genome-wide SNP data including 37 k markers allowed investigators to derive more accurate relatedness than incomplete pedigrees, leading to both less-biased heritability estimates and improved separation of direct genetic effects from maternal components (22). Pig data collected with 60 k SNP showed that carefully constructed genomic relationship matrices yielded high correlations to true genome sharing, translating to more precise breeding values than those obtained from pedigree alone (23). The benefits of dense genomic information for low-heritability traits are further highlighted in a commercial rainbow trout population, where gEBV for disease resistance far exceeded pedigree-based BLUP and allowed breeders to exploit within-family variation (24). Additionally, attempts to implement selection programs in smaller sheep flocks demonstrated that pedigree-only methods were hampered by missing sire information and limited accuracy. In contrast, adding genomic information offered at least modest improvements that were more apparent as flock size increased (25). These findings suggest that pedigree-based selection alone may not fully capture the subtle genetic architecture of complex traits. By contrast, genomic breeding values incorporate realized genomic relationships, allowing for a more precise estimation of genetic merit in populations with low heritability, high QTL counts, or incomplete pedigrees. Consequently, this paper focuses on genomic selection to address the inherent constraints of traditional approaches, thereby improving the reliability of breeding decisions for complex traits.

### 4.2 Performance of individual trait genomic EBVs

The findings of this study provide a critical evaluation of the potential for genomic selection methodologies, particularly Genomic Best Linear Unbiased Prediction (GBLUP), to enhance guide dog breeding programs. While the effectiveness of GBLUP has been well-established in livestock breeding, its application to working dog populations remains largely unexplored. This study sought to develop a multi-trait selection index integrating health and behavioral metrics to optimize breeding decisions for guide dog success. The results highlight both the opportunities and limitations of genomic selection in this complex context, with broader implications for reference population construction and breeding value estimation in novel populations.

To begin, individual trait breeding values were generated and assessed for predictive performance. Individual trait gEBV provides a more targeted selection of specific traits. The predictive accuracy of gEBV varied across individual traits, with dentition-related conditions, such as mandibular distocclusion, malocclusion class I, and mandibular mesiocclusion, exhibiting a generally higher predictive performance. The estimated heritabilities aligned with the model performance with generally higher predictive power for more heritable traits. These results suggest that certain health traits have a strong genetic basis and may be more immediately improved through genomic selection. We expect these dental traits to respond similarly to genetic selection through EBVs, as seen with the decrease in hip dysplasia prevalence in guide dogs (7–9). However, other conditions, including dermatological and neurological traits, showed weaker predictive accuracy, indicating a lower genetic heritability or significant environmental influences. Another potential limitation is the markers used within the study, which may not capture all variation in the breeds of interest. The purpose of commercial arrays are to capture variation across all breeds in a cost-effective manner but are still limited by SNP density and array design. Despite this potential limitation, previous work did not identify significant increases in GBLUP predictive performance with increased marker density in guide dog gEBV model performance (12). More specialized breed-specific genotyping has been implemented in cattle to alleviate this specific problem, but it would require more investment to implement in guide dog populations (26). Regardless, these traits may still be strong candidates for selection as genomic selection sees the most improvement compared to pedigree-based selection in less heritable, more complex to predict traits when genomic marker association is available (27).

Most behavioral traits were difficult to predict accurately using genetic data. Correlations were generally weak, particularly for traits like soundness and trainability, highlighting the challenge of linking complex behaviors to genetics. The weak correlations observed for most behavioral traits, especially soundness and trainability, reflect a broader challenge in canine genetics: the difficulty of reliably predicting complex behavioral phenotypes from genetic data alone. Across all traits, MCC was low to 0, indicating that the unbalanced nature of the datasets leads to poor predictive accuracy even if the AUROC shows that risk ranking is more useful. The exception to lower behavioral trait performance and the only one with an MCC score above 0 was distraction, which exhibited the highest predictive accuracy of all behavioral traits and may reflect increased consistency in the rating of this trait and heritability.

Because inclusion required each dog to reach the mid-term blindfold assessment (~6–8 weeks into training), our analysis necessarily omits any animal eliminated by the initial health or temperament screens. These limitations mean that severe, early-onset diagnoses that guarantee rejection do not appear in the dataset and the health phenotypes we do have are almost all incidental findings noted during routine physical exams. These conditions are far less informative than the six behavioral scores that instructors assign at the mid-term and, for dogs that progress further, again at the final blindfold test (≈12–14 weeks). This pipeline produces a cohort already biased toward eventual success, so the traits we examine are precisely those that remain hard to filter out before substantial resources have been committed to raising, caring for, and training each dog. Including these early failure dogs would have introduced imbalance for the composite indices as the animals would lack most behavior and health data collected later in training. The early removal of animals would leave incomplete trait profiles making them, categorically different, and less comparable to those of dogs who progressed far enough for structured assessment. For future application purposes, each trait will include all available dogs with the respective trait data. This will likely improve trait prediction accuracy but would not have provided comparable analysis across traits for the purpose of this research.

In summary, many traits showed MCC values of 0.000, indicating poor classification performance in an unbalanced dataset, yet some (e.g., mandibular distocclusion, malocclusion class I, mandibular mesiocclusion) exhibited relatively high AUROC scores (0.785–0.865), suggesting that while the model struggled to set an optimal threshold for binary classification, it still demonstrated useful ranking ability. Moreover, traits such as anodontia had an MCC greater than zero (0.084), highlighting a marginal but notable capacity for classification. These mixed results underscore the complexity of leveraging GBLUP for binary classification and are an example of the potential strengths of ranking risk compared with correct categorization. These results suggest that even with an unbalanced dataset of health traits, risk can still be a viable metric to allow for the utilization of breeding values.

### 4.3 Performance of targeted and total score indices

A key insight from this study is the greater correlation of behavioral traits compared with health traits in determining guide dog success in our dataset of animals that passed initial health assessments. The lower performance of health-based indices as fixed effects to improve the model’s ability to predict success suggests that genetic predisposition to specific health conditions does not necessarily preclude a dog from successfully completing training. This outcome is likely reflective of the health traits included in this dataset due to limiting the dogs included based upon their behavior testing timeline. Instead, behavioral attributes such as the behavior score and total score weighted for behavior showed stronger associations with guide dog outcomes as fixed effects. In particular, success had an AUROC of 0.570 when predicted alone. However, including the behavior score as a fixed effect raised the AUROC to 0.718 and yielded an MCC of 0.212, emphasizing the importance of specific behavioral traits in determining training completion.

The moderate performance of the breeding values to predict the behavior score index and total score index means that the fixed effects for success may need to rely more on phenotypic values as opposed to being able to use gEBVs as the fixed effects for earlier determination before phenotypic records are collected. These findings parallel recent efforts in service dog breeding programs, where temperament and trainability are recognized as more important selection criteria than physical health alone (4, 28). This underscores the need for multi-trait selection frameworks that place greater weight on behavioral indicators while considering health-related risk factors to help prevent unintentional negative selective pressures. Although the dairy industry has led the way in genomic improvement, this lesson about the importance of monitoring and selecting for all desired traits, not only ones highly correlated to a “successful” animal, comes from the negative correlation between reproductive and production traits. After selecting heavily for production traits for decades, a decline in fertility led to changing selective weights to incorporate selection for reproductive traits to correct the decline in fertility (29, 30).

Notably, many binary health traits had MCC values at or near zero, reflecting limited strict classification utility in this unbalanced dataset; however, some dentition conditions, e.g., mandibular distocclusion AUROC = 0.865, malocclusion class I AUROC = 0.790, mandibular mesiocclusion AUROC = 0.785, and anodontia AUROC = 0.706, MCC = 0.084 exceeded an AUROC threshold of ~0.70, supporting risk-ranking value in a breeding program. By contrast, supernumerary teeth AUROC = 0.453 and numerous dermatological conditions such as muzzle folliculitis AUROC = 0.527 and acral lick dermatitis AUROC = 0.534 offered weaker predictive discrimination. Similarly, composite metrics like Dental Count showed a modest correlation (Kendall’s Tau = 0.197, Spearman’s Rho = 0.247), but Dermal Count was essentially uninformative Kendall’s Tau = −0.020, Spearman’s Rho = −0.025. While the overall Health Score exhibited only low correlations (Pearson’s R ~ 0.1), the Behavior Score and Total Score indices performed better, both as individual targets (Pearson’s R ~ 0.23) and especially when used as fixed effects for predicting success (AUROC ~0.72). Based on these findings, breeding programs can implement dentition EBVs and behavior and total score indices immediately using top-quartile or other clear ranking cutoffs. In contrast, traits with AUROC values below ~0.65 will likely require larger datasets or refined phenotyping before they can substantively guide selection.

### 4.4 Study limitations

The reference population constrained the effectiveness of genomic selection in this study, an issue widely recognized in other species (3, 31). The accuracy of breeding values is inherently tied to the reference population’s size, diversity, and representativeness. In livestock, where genomic selection has been most successful, large, multi-institutional datasets have provided extensive training populations for genomic prediction (2, 30). In contrast, guide dog breeding programs typically operate with relatively small, closed populations. This leads to reduced genetic variance and limited selection intensity due to the lack of diverse variants for associations to be identified (10). This study’s modest predictive power of health and behavioral indices suggests that the reference population may not yet be large or genetically diverse enough to fully capture the genetic variation underlying some key guide dog traits, particularly of less related individuals from other organizations. This finding is consistent with the idea that large populations are necessary for training datasets, and as the diversity of the target population increases, the reference population must expand to match.

Another challenge lies in the subjectivity and standardization of behavioral assessments. Unlike physical health traits, which can often be more objectively measured, many behavioral traits rely on trainer evaluations, which may introduce observer bias and variability. While efforts were made to standardize assessments in this study, such as the use of only a few evaluators to minimize interrater discrepancies, differences in handler interpretation and environmental contexts may have contributed to inconsistencies in trait expression, a challenge recognized in previous research on canine behavioral genetics (32). Within organizational breeding programs, this variability may be adjusted for. However, for optimal integration of breeding values across organizations, this remains a significant challenge. Future studies may also consider more objective measures of behavior, such as biometric and neurophysiological markers, to enhance the precision of behavioral trait assessments (33, 34).

### 4.5 Future application

Despite these challenges, this study highlights several opportunities for advancing genomic selection in guide dog breeding. The moderate predictive performance of behavioral indices suggests that genomic data can contribute to selection decisions, albeit with some consideration for the population and fixed effects included. Expanding genotypic and phenotypic datasets will also be critical. Increasing sample sizes and collecting additional behavioral, health, and physiological phenotypes may enhance the robustness of genomic selection models.

Additionally, collaborations between guide dog and other dog breeding organizations could provide access to larger, more genetically diverse populations, improving statistical power and enhancing the reliability of selection indices. A coordinated effort across multiple dog breeding programs would accelerate genetic improvement efforts and create a more comprehensive genomic database for working dogs. The possibility of including AI models and biometric data for phenotypic data also offers a roadmap from livestock that could be adopted to address the challenges of combining phenotypic data from multiple organizations (35).

This study demonstrates the significant potential of genomic selection methodologies, particularly Genomic Best Linear Unbiased Prediction (GBLUP), to improve the efficiency and accuracy of breeding decisions in guide dog programs. Integrating multi-trait selection indices incorporating health and behavioral attributes provides a scientifically rigorous framework for enhancing genetic gain, selection efficiency, and overall breeding outcomes.

Our findings highlight the greater predictive power of behavioral traits over most health conditions in determining guide dog success, reinforcing the necessity of incorporating behavior into selection models. These findings must be considered with the caveat that the included health traits did not include severe, early-onset diagnoses that may cause dismissal before behavioral assessment. Among health conditions, dental abnormalities exhibited the strongest genetic predictability, while dermatological traits showed weaker associations, likely due to environmental influences. Including parental fixed effects improved predictive accuracy, supporting the value of multi-generational genomic data in selection decisions.

Despite these advancements, challenges remain. The relatively low predictive power of some binary health traits and the need for standardized phenotypic assessments present ongoing hurdles for genomic selection in guide dog breeding. Future research should focus on expanding genomic reference populations and refining behavioral and health trait phenotyping across organizations.

By leveraging genomic technologies and multi-trait selection indices, guide dog breeding programs can systematically enhance selection accuracy, reduce hereditary conditions’ prevalence, and improve working dogs’ success rate. As genomic tools continue to evolve, their integration into breeding strategies will be crucial for ensuring the long-term health, performance, and sustainability of guide dog populations, ultimately improving the lives of both the dogs and the individuals they serve.

## Data Availability

The data analyzed in this study is subject to the following licenses/restrictions: the datasets presented in this article are not available in a data repository as they are owned by the Seeing Eye. Author, Katy M. Evans, may be contacted to inquire about access to and use of the datasets. Requests to access these datasets should be directed to Katy M. Evans, kevans@seeingeye.org, The Seeing Eye.

## Footnotes

^1^ www.goldenhelix.com
